# Actinobacteria Isolated From *Laminaria ochroleuca*: A Source of New Bioactive Compounds

**DOI:** 10.3389/fmicb.2019.00683

**Published:** 2019-04-09

**Authors:** Mariana Girão, Inês Ribeiro, Tiago Ribeiro, Isabel C. Azevedo, Filipe Pereira, Ralph Urbatzka, Pedro N. Leão, Maria F. Carvalho

**Affiliations:** ^1^Interdisciplinary Centre of Marine and Environmental Research, University of Porto, Porto, Portugal; ^2^Department of Biology, Faculty of Sciences, University of Porto, Porto, Portugal

**Keywords:** marine actinobacteria, endophytic actinobacteria, bioactivity, antimicrobial, anticancer, macroalgae, kelp, *Laminaria ochroleuca*

## Abstract

Nature is the major reservoir of biologically active molecules. The urgent need of finding novel molecules for pharmaceutical application is prompting the research of underexplored environments, such as marine ecosystems. Here, we investigated cultivable actinobacteria associated with the macroalgae *Laminaria ochroleuca* and assessed their potential to produce compounds with antimicrobial or anticancer activities. A specimen of *L. ochroleuca* was collected in a rocky shore in northern Portugal, and fragments of tissues from different parts of the macroalgae (holdfast, stipe, and blades) were surface sterilized and plated in three culture media selective for actinobacteria. A total of 90 actinobacterial strains were isolated, most of which affiliated with the genus *Streptomyces*. Isolates associated with the genera *Isoptericola*, *Rhodococcus*, *Nonomuraeae*, *Nocardiopsis*, *Microbispora*, and *Microbacterium* were also obtained. Organic extracts from the isolates were tested for their antimicrobial activity using the agar-based disk diffusion method, followed by determination of minimum inhibitory concentration (MIC) values. Forty-five isolates inhibited the growth of *Candida albicans* and/or *Staphylococcus aureus*, with MIC values ranging from <0.5 to 1000 μg mL^−1^. The actinobacterial isolates were also tested for their anticancer potential on two human cancer cell lines. Twenty-eight extracts affected the viability of at least one human cancer cell line (breast carcinoma T-47D and neuroblastoma SH-SY5Y) and non-carcinogenic endothelial cell line (hCMEC/D3). Seven extracts affected the viability of cancer cells only. This study revealed that *L. ochroleuca* is a rich source of actinobacteria with promising antimicrobial and anticancer activities and suggests that macroalgae may be a valuable source of actinobacteria and, consequently, of new molecules with biotechnological importance.

## Introduction

Natural products (NP) play an important role in several sectors of our society. These compounds are valuable from industrial, biotechnological and pharmacological perspectives. They have been instrumental in combating diverse human diseases, such as cancer and bacterial infections (Harvey, 2008). From 1940 to 2014, about half of the approved drugs for use in cancer therapy were either NP or NP-inspired or -derived compounds (Newman and Cragg, 2016). Microorganisms produce a wide diversity of NP that translates in a wide bioactivity range (Berdy, 2005). This is particularly noteworthy for the phylum Actinobacteria, and more so in the homonym class, commonly known as actinomycetes and hereafter referred to as actinobacteria. In fact, bioactivities reported from actinobacterial NP include antibacterial, antifungal, antitumor, anticancer, anti-inflammatory, antiviral, cytotoxic, and immunosuppressive activities (Dharmaraj, 2010; Manivasagan et al., 2014; ul Hassan and Shaikh, 2017). So far, more than 10,000 different bioactive molecules have been reported from actinobacteria, representing nearly 45% of the bioactive microbial metabolites currently known, the majority of which having been isolated from the genus *Streptomyces* (Berdy, 2005; Azman et al., 2017). For this reason, in the last decades, multiple NP discovery efforts have focused on actinobacteria, especially those from terrestrial sources. However, the discovery of novel compounds from terrestrial actinobacteria is reaching a stagnation point, impelling the scientific community to look for new chemical diversity in much less explored environments, such as marine ecosystems (Bhatnagar and Kim, 2010). Marine actinobacteria possess unique physiological adaptations to conditions very distinct from those in terrestrial environments, namely in terms of pressure, salinity and temperature (Undabarrena et al., 2016). These adaptations are also reflected in a differentiated secondary metabolome (Abdelmohsen et al., 2015). Two notable illustrations of the potential of marine actinobacteria to produce novel lead drugs are salinosporamide A, produced by the marine actinobacteria *Salinospora tropica*, now in clinical trials to treat patients with multiple myeloma, solid tumors, and lymphoma (Pérez and Fenical, 2017), and abyssomicin C, isolated from a marine *Verrucosispora*, exhibiting antibacterial activity against methicillin-resistant *Staphylococcus aureus* (MRSA) and *Mycobacterium tuberculosis* (Bister et al., 2004; Freundlich et al., 2010).

Actinobacteria are widely distributed in the marine environment, in particular in sediments, but they also exist in association with a number of marine organisms, such as fishes, sponges, macroalgae, corals, and tunicates (Abdelmohsen et al., 2014). Endophytic microorganisms live in the inner tissues of plants and algae without causing negative damages to the host (Petrini, 1991). During this symbiotic association, endophytes produce secondary metabolites that improve the fitness of the host and its resistance against environmental stressors, obtaining in return nutrients and shelter from their host (Dinesh et al., 2017). Macroalgae are known to host diverse species of actinobacteria, both epiphytic and endophytic, but little has been done to assess their biotechnological potential, with the majority of the bioactivity screenings targeting epiphytic microorganisms, mainly fungi (Egan et al., 2008). Nevertheless, some studies demonstrated that actinobacteria isolated from macroalgae are capable of producing bioactive compounds, including antibiotics, antitumor, and anti-inflammatory compounds (Braña et al., 2015; Uzair et al., 2018).The brown alga *Laminaria ochroleuca* forms complex structures, known as kelp forests, which dominate shallow rocky shores of cold-water marine habitats worldwide (Franco et al., 2018). Kelp forests are among the most diverse and productive ecosystems in the world (Steneck et al., 2002). *L. ochroleuca* is an abundant and ecologically-important species of macroalga (Franco et al., 2018), but its associated actinobacterial diversity was hitherto uncharacterized. Here, we isolated culturable actinobacteria from *L. ochroleuca*, and investigated their antimicrobial and anticancer potential.

## Materials and Methods

### Sampling and Bacterial Isolation

One specimen of *L. ochroleuca* was collected in the intertidal area of the rocky shore at Mindelo, located in northern Portugal (41.309298°; −8.742228°). The alga was transported to the laboratory under refrigeration and processed on the same day. The collected specimen was washed with sterile sea water and segmented into three distinct parts: holdfast, stipe and blades. Each part was cut into approximately 2 cm long pieces that were incubated in a cetyl trimethylammonium bromide (CTAB) buffer solution (diluted 1:100) containing proteinase K (20 mg mL^−1^) for 30 min at 60°C, and washed for three consecutive times with sterile sea water for 1 min (Hollants et al., 2010). The effectiveness of the surface sterilization was evaluated by plating the final wash water and sterilized pieces from each part of the algae on Plate Count Agar (PCA) (Liofilchem, Roseto d. Abruzzi, Italy). Algae pieces were ground and inoculated on selective culture media, namely Starch-Casein-Nitrate agar (SCN: 10 g of soluble starch, 0.3 g of casein, 2 g of K_2_HPO_4_, 2 g of KNO_3_, 2 g of NaCl, 0.05 g of MgSO_4_.7H_2_O, 0.02 g of CaCO_3_, 0.01 g of FeSO_4_.7H_2_O, and 17 g of agar, per liter of distilled water), Raffinose-Histidine agar (RH: 10 g of raffinose, 1 g of L-Histidine, 1 g of K_2_HPO_4_, 0.5 g of MgSO_4_.7H_2_O, 0.01 g of FeSO_4_.7H_2_O, and 17 g of agar, per liter of distilled water) and Nutrient-Poor Sediment Extract agar (NPS: 100 mL of marine sediment extract obtained by washing 900 mL of sediments with 500 mL of seawater and 17 g of agar, per liter of seawater). The culture media were supplemented with cycloheximide (50 mg L^−1^; Sigma-Aldrich, MO, United States) and nalidixic acid (50 mg L^−1^; AppliChem, Darmstadt, Germany) to inhibit the growth of fungi and Gram-negative bacteria, respectively. The plates were incubated at 28°C for a period of up to 6 weeks. Morphologically distinct colonies were isolated and preserved at −80°C in 30% (v/v) of glycerol. The colonies were named using the “K” code, indicative of kelp, “EN” indicative of endophyte, and “R”, “B”, and “S” for colonies isolated from the holdfast (rhizoid), blade, and stipe fragments, respectively (Supplementary Table S1).

### Taxonomic Identification of the Obtained Isolates

All isolates were taxonomically identified through 16S rRNA gene sequencing. For each isolate, genomic DNA was extracted using the E.Z.N.A.^®^ Bacterial DNA Kit (Omega Bio-Tek, GA, United States) according to the manufacturer’s instructions. The 16S rRNA gene was amplified by PCR using the universal primers 27F (5′-GAGTTTGATCCTGGCTCAG-3′) and 1492R (5′-TACGGYTACCTTGTTACGACTT-3′) (Stackebrandt and Goodfellow, 1991; Weisburg et al., 1991). The PCR mixture (final volume of 10 μL) consisted of: 5 μL of Qiagen Multiplex PCR Master Mix (Qiagen, CA, United States), 1 μL of each primer (2 μM), 2 μL of DNA template and 1 μL of nuclease-free water. The reaction was started with an initial denaturation at 95°C for 15 min followed by 30 cycles of denaturation at 94°C for 30 s, annealing at 48°C for 90 s and extension at 72°C for 90 s, and a final extension at 72°C for 10 min. PCR products were separated on a 1.4% agarose gel containing SYBR Safe (Thermo Fisher Scientific, MA, United States). PCR products were purified with ExoSAP-IT (TM) Express (Applied Biosystems, Warrington, United Kingdom), a one-step enzymatic treatment to eliminate unincorporated primers and dNTPs, according to manufacturer’s recommendations. Sequencing of purified samples (2.5 μL) was performed in a Genetic Analyzer 3130xl sequencer (Applied Biosystems, Warrington, United Kingdom), according to the manufacturer’s recommendations. The obtained 16S rDNA sequences were analyzed using the Geneious software, version 11.1.4. (Biomatters, Auckland, New Zealand). The taxonomic affiliation of the isolates was established using the 16S ribosomal RNA (Bacteria and Archaea) database from NCBI BLAST tool^1^ and confirmed using the identify tool from EzTaxon^2^ and the sequence match tool from the Ribosomal Database Project^3^. To complement the taxonomic study of the isolates, a phylogenetic tree was elaborated. According to BLAST results, the closest neighbor sequences in GenBank for each isolate were selected, with no more than a single sequence being selected for the same species. A total of 182 sequences that fulfilled these criteria were retrieved for all isolates and these were aligned using MUSCLE from within the Geneious software package. The phylogenic tree was then constructed from this alignment, using the Maximum Likelihood method with 1000 bootstraps based on the Tamura-Nei model. Strains KENR90, 91 and 92 were not included in the tree since their nucleotide sequences were too short (590, 617, and 833 bp, respectively). The tree was constructed using the Molecular Evolutionary Genetics Analysis program Version 7.0 (MEGA7) (Kumar et al., 2016).

### Preparation of Crude Extracts for Bioactivity Assays

Each actinobacterial isolate was grown in a 100 mL Erlenmeyer flask containing 30 mL of liquid culture medium with a composition identical to the solid medium from which the isolate was obtained, but without the addition of cycloheximide and nalidixic acid. The flasks were incubated at 28°C, 100 rpm, in the dark. After 4 days of incubation, 0.5 g of Amberlite XAD16N resin (Sigma-Aldrich, MO, United States) were added to the culture medium and incubation continued for three additional days. Each culture was extracted together with the resin by adding 30 mL of a solution of acetone/methanol 1:1 (v/v). The organic layer was recovered and dried in a rotary evaporator to yield an organic extract that was weighted before being dissolved in dimethyl sulfoxide (≥99.9%, DMSO; Sigma-Aldrich, MO, United States) to prepare stock solutions of 10, 3.0, and 1.0 mg mL^−1^, which were used for the bioactivity assays.

### Screening of Antimicrobial Activity

The antimicrobial activity of the crude extracts obtained from the actinobacterial isolates was tested against five reference microorganisms - *Escherichia coli* (ATCC 25922), *Bacillus subtilis* (ATCC 6633), *Staphylococcus aureus* (ATCC 29213), *Salmonella typhimurium* (ATCC 25241), and *Candida albicans* (ATCC 10231) – using the agar-based disk diffusion method (Balouiri et al., 2016). The bacterial strains were grown in Mueller-Hinton agar (MH) and *C. albicans* in Sabouraud Dextrose agar (SD) (Liofilchem, Roseto d. Abruzzi, Italy). For the bioassay, reference microorganisms were suspended in the corresponding liquid medium and their turbidity was adjusted to 0.5 McFarland standard (OD_625_ = 0.08-0.13). The suspensions were used to inoculate agar plates (MH or SD, according to the reference microorganism) by evenly streaking the plates with a swab dipped in the suspension. Blank paper disks (6 mm in diameter) were placed on the surface of the inoculated medium and 15 μL of each actinobacterial crude extract (1000 μg mL^−1^) were loaded into the disks. Positive control disks were inoculated with 15 μL of enrofloxacin (1000 μg mL^−1^; Sigma-Aldrich, MO, United States) for the bacterial strains, and 15 μL of nystatin (1000 μg mL^−1^; Sigma-Aldrich, MO, United States) for *C. albicans*, while negative control disks were inoculated with 15 μL of DMSO. One replica of each extract was tested in two independent experiments. Antimicrobial activity was determined by measuring the diameter of the inhibition halo formed around each disk after 24 h of incubation at 37°C. For extracts exhibiting antimicrobial activity, the minimum inhibitory concentration (MIC) was also determined. In the context of this study, MIC is defined as the lowest extract concentration that completely inhibits growth of a test microorganism. For MIC determination, inoculum suspensions of the reference strains were prepared as mentioned above. For each extract, stock solutions at 1000 μg mL^−1^ were prepared in the appropriate culture medium. Two-fold dilutions in the same medium were sequentially obtained from these stocks, resulting in extracts solutions with concentrations ranging from 1000 to 0.5 μg mL^−1^. The assay was conducted in 96 well plates by adding 50 μL of the microbial inoculum (diluted 1:100) and 50 μL of each extract dilution to each well. The MIC was determined by spectrophotometric analysis (625 nm; model V-1200, VWR, PA, United States), after 18 h of incubation at 37°C. The positive growth control consisted in 50 μL of microbial inoculum and 50 μL of medium broth and the negative growth control in 100 μL of medium broth. For each extract, MIC was determined in triplicate and two independent experiments were performed.

### Screening of Anticancer Activity

T-47D cells (breast ductal carcinoma) were grown in Dulbecco’s Modified Eagle Medium (DMEM) (Gibco, Thermo Fischer Scientific, Waltham, MA, United States) and SH-SY5Y cells (neuroblastoma) in a 1:1 mixture of MEM/F12 medium, both supplemented with 10% (v/v) fetal bovine serum (Biochrom, Berlin, Germany), 1% (v/v) antibiotics (100 mg L^−1^ streptomycin), 100 IU mL^−1^ penicillin (Biochrom, Berlin, Germany) and 0.1% (v/v) amphotericin (GE Healthcare, Little Chalfont, United Kingdom), at 37°C in an incubator with 5% carbon dioxide. The anticancer activity of each actinobacterial crude extract was tested in both human cancer cell lines using the MTT [3-(4,5-dimethylthiazol-2-yl)-2,5-diphenyltetrazolium bromide] assay. The cells were seeded in 96-well plates at 6.6 × 10^4^ cells mL^−1^, allowed to adhere overnight and exposed to the extracts (final concentration of 15 μg mL^−1^, 1:200 dilution of 3 mg mL^−1^ stock solution) for 24 and 48 h. The positive control consisted of 20% DMSO (a concentration known to be toxic to the majority of cells within 24 h) and the solvent control in 0.5% DMSO (same solvent concentration as crude extracts). After 24 and 48 h of exposure, 20 μL of MTT (final concentration: 0.2 mg mL^−1^; Sigma-Aldrich, MO, United States) were added per well and the plates were incubated for an additional period of 4 h at 37°C. After this step, the culture medium was removed from each well and 100 μL of DMSO was added to the wells. The absorbance at 570 nm in each well was determined in a multimode plate reader (model Synergy HTX, Biotek, VT, United States). Two independent experiments were performed for each cell line. In each experiment the extract was tested in triplicate. Cellular viability was expressed as a percentage relative to the solvent control. The extracts exhibiting anticancer activity were additionally tested against a non-tumor cell line (human brain capillary endothelial cells, hCMEC/D3, kindly donated by Dr. P. O. Courad, INSERM, France) to evaluate their general cytotoxicity, following the procedures described above.

### Statistical Analysis

Data from anticancer assays (a total of six technical replicates) was tested for significant differences compared to the solvent control, and the significance level was set for all tests at *p* < 0.05. The Kolmogorov Smirnov test was used to check for normality distribution of data. For parametric data, one-Way ANOVA was applied followed by Dunnett’s *post hoc* test. If statistical test assumptions were not met, data were either square root transformed and re-tested by ANOVA, or the non-parametric Kruskal-Wallis test was applied, followed by Dunn’s multiple comparison test.

### Dereplication and Molecular Network Analysis

A set of 35 extracts were selected from those exhibiting highest activities (inhibition halos >1 cm in the antimicrobial assays and/or those that were able to inhibit >70% of cell proliferation compared to the solvent control in the cancer cell line cytotoxicity assays). These extracts were resuspended in methanol at a concentration of 2 mg mL^−1^ and used for LC-HRESIMS/MS analysis on a system composed of a Dionex Ulimate 3000 HPLC coupled to a qExactive Focus Mass spectrometer controlled by XCalibur 4.1 software (Thermo Fisher Scientific, MA, United States). Ten microliters of each extract were injected into an ACE UltraCore 2.5 Super C18 (75 mm × 2.1 mm) column (Advanced Chromatography Technologies, Aberdeen, United Kingdom). The separation was carried out using a gradient from 99.5 to 10% water/methanol/formic acid (95:5:0.1, v/v) to 0.5 to 90% isopropanol/methanol/formic acid (95:5:0.1, v/v) for 9.5 min and held for 6 min before returning to the initial conditions. The UV absorbance of the eluate was monitored at 254 nm and a Full MS scan at the resolution of 70,000 FWHM (range of 150–2000 m/z), and data dependent MS^2^ (ddMS^2^, Discovery mode) at the resolution of 17,500 FWHM (isolation window used was 3.0 amu and normalized collision energy was 35). Raw data files obtained from these analyses were converted to the mzML format and used in the dereplication workflow of the Global Natural Products Social Molecular Networking platform (GNPS) (Wang et al., 2016). Nine extracts resulted in no hits in the dereplication analysis that could explain the observed activities. These were submitted to the GNPS data analysis workflow using default parameters (except ion mass tolerance precursor, which was set to 0.01 Da and fragment ion mass tolerance, which was set at 0.04 Da, to account for high-resolution data). The resulting molecular network was visualized with Cytoscape v3.6.1 (Shannon et al., 2003) and searched for clusters of m/z data that were associated with a single extract. The HRESIMS/MS spectra corresponding to such data were annotated to clarify the likely masses of the compounds generating the clusters. These putative compound masses were used for an additional dereplication step by searching the predicted accurate mass against the Dictionary of NP database (version 27.1). When hits from Actinobacteria were obtained, information regarding the biological activity of the compounds was retrieved from this database.

## Results

### Isolation and Identification of Actinobacteria From *L. ochroleuca*

**FIGURE 1:**
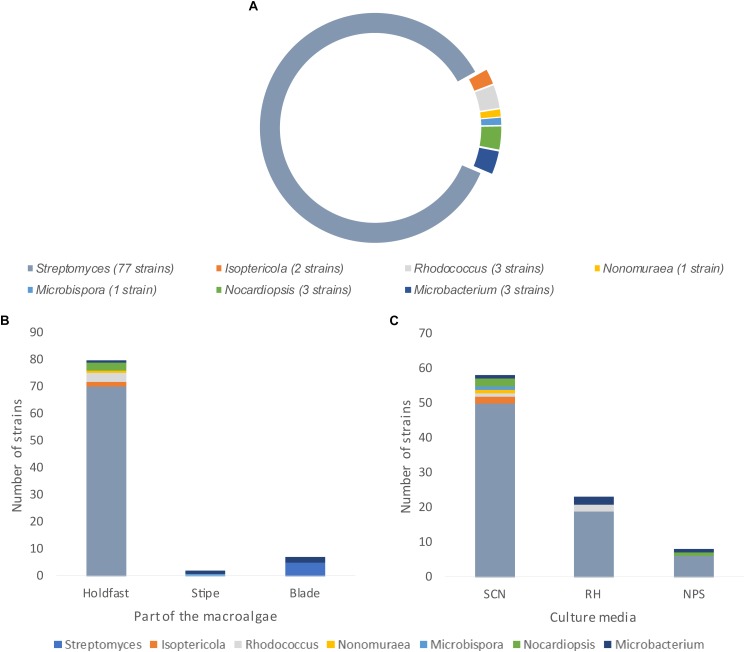
Actinobacterial genera recovered from *L. ochroleuca*. **(A)** Number of actinobacterial strains affiliated with the different genera retrieved from *L. ochroleuca*, **(B)** genera distribution in the holdfast, stipe, and blades of the macroalgae and **(C)** genera distribution according to the selective culture media used for the isolation.

**FIGURE 2:**
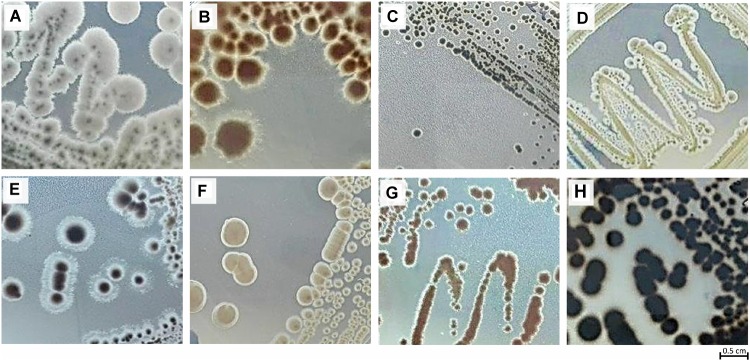
Morphological diversity of some actinobacterial strains isolated from *L. ochroleuca*: **(A)** strain KENS1, **(B)** strain KENR91, **(C)** strain KENR92, **(D)** strain KENR94, **(E)** strain KENB10, **(F)** strain KENR56, **(G)** strain KENR81, and **(H)** strain KENB8.

**FIGURE 3:**
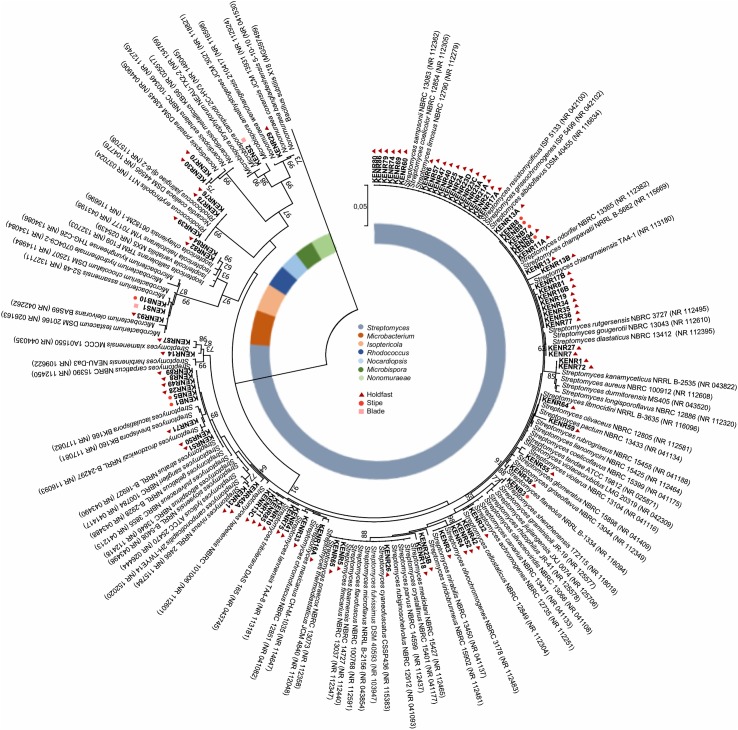
Phylogenetic tree (16S rRNA gene) obtained by Maximum Likelihood analysis of the 87 actinobacterial isolates recovered from *L. ochroleuca*, together with a selection of closely related strains with sequences deposited in GenBank. The tree was generated using 1281 bp and 1000 bootstraps. Numbers at nodes represent bootstrap values (%) when higher than 60%. Numbers in parenthesis correspond to GenBank accession numbers. *Bacillus subtilis* was used as an outgroup.

A total of 90 actinobacterial strains were isolated from sterilized tissue fragments obtained from the holdfast, stipe, and blades of *L. ochroleuca* (Figure 1A). Actinobacterial strains were isolated from all parts of the macroalgae (Figure 1B) and recovered from the three selective culture media used (Figure 1C). Several strains presented typical characteristics of actinobacteria, such as slow growth, formation of hyphae and production of spores and pigments, the latter sometimes leading to a change of the color of the culture medium (Figure 2). The sterilization treatment was not entirely effective, as five isolates were recovered from the sterilization controls. Two of such isolates, identified as *Pseudomonas* sp. and *Lysinibacillus* sp., were obtained from a holdfast fragment, two were obtained from the final wash water of a holdfast fragment and identified as *Bacillus* sp. and *Staphylococcus* sp., and one was derived from the final wash water of a blade fragment and identified as *Penicillium* sp. *Streptomyces*, *Isoptericola*, *Rhodococcus*, *Nonomuraeae*, *Nocardiopsis*, and *Microbacterium* strains were isolated from the holdfast, *Microbispora* and *Microbacterium* strains from the stipe, and *Streptomyces* and *Microbacterium* strains from the blades (Figure 1A,B, 3). The selective medium SCN allowed the retrieval of strains belonging to all genera identified in this study (Figure 1C). Growth of *Streptomyces* and *Microbacterium* strains was observed in all culture media used. The evolutionary relationships between the isolated actinobacterial strains was inferred from a 16S rRNA gene phylogenetic tree (Figure 3). Several of the isolated strains, many of them affiliated with the genus *Streptomyces*, were found to group very closely in a well-supported clade, which indicates a high similarity between these strains.

### Antimicrobial and Anticancer Potential of the Obtained Actinobacterial Isolates

Antimicrobial assays identified 45 isolates capable of inhibiting the growth of *C. albicans* (*n* = 37), *S. aureus* (*n* = 3) or both (*n* = 5). These isolates were affiliated with the genera *Streptomyces* (39 strains), *Isoptericola* (one strain), *Nonomuraeae* (one strain), *Nocardiopsis* (one strain), *Microbispora* (one strain), and *Microbacterium* (two strains). The diameters of inhibition halos varied from 0.7 to 2.5 cm (Supplementary Figure S1) and the determined MIC values ranged between <0.5 and 1000 μg mL^−1^ (Table 1). The MIC value was reproduced once. The lowest antibacterial MIC value was determined for *S. aureus* (3.9 μg mL^−1^) exposed to extracts of the *Streptomyces* strains KENR60 and 64, isolated from the holdfast. In turn, the lowest antifungal MIC value (<0.5 μg mL^−1^ against *C. albicans*) was observed for the crude extract of *Streptomyces* KENR25, also isolated from the holdfast.

**Table 1:** Actinobacteria isolated from *L. ochroleuca* with antimicrobial activity.

		Diameter of Inhibition Halos		MIC (μg mL^−1^)	
ISOLATE	TAXONOMIC IDENTIFICATION	*S. aureus*	*C. albicans*	*S. aureus*	*C. albicans*
KENR3	*Streptomyces olivochromogenes*			n.d.	31.3
KENR6	*Streptomyces* sp.			1000	1.0
KENR8	*Streptomyces atratus*			n.d.	2.0
KENR11A	*Streptomyces* sp.			n.d.	7.8
KENR13	*Streptomyces* sp.			n.d.	2.0
KENR13A	*Streptomyces* sp.			n.d.	1.0
KENR13B	*Streptomyces* sp.			1000	47.8
KENR14	*Streptomyces iamenensis*			n.d.	7.8
KENR16B	*Streptomyces* sp.			n.d.	15.6
KENR17A	*Streptomyces* sp.			n.d.	2.0
KENR18	*Streptomyces* sp.			1000	1.0
KENR19	*Streptomyces* sp.			n.d.	1.0
KENR21	*Streptomyces* sp.			n.d.	2.0
KENR21A	*Streptomyces* sp.			n.d.	1.0
KENR24	*Streptomyces* sp.			n.d.	7.8
KENR25	*Streptomyces* sp.			n.d.	<0.5
KENR29	*Nonomuraea* sp.			n.d.	3.9
KENR31	*Streptomyces* sp.			62.5	1000
KENR33	*Streptomyces lannensis*			n.d.	2.0
KENR34	*Streptomyces* sp.			n.d.	15.6
KENR35	*Streptomyces* sp.			n.d.	1.0
KENR36	*Streptomyces* sp.			n.d.	125
KENR42	*Streptomyces mirabilis*			n.d.	125
KENR49	*Streptomyces atratus*			n.d.	3.9
KENR60	*Streptomyces* sp.			3.9	n.d.
KENR64	*Streptomyces* sp.			3.9	n.d.
KENR65	*Streptomyces* sp.			n.d.	125
KENR69	*Streptomyces* sp.			n.d.	15.6
KENR70	*Nocardiopsis prasina*			n.d.	15.6
KENR72	*Streptomyces aureus*			n.d.	2.0
KENR74	*Streptomyces* sp.			n.d.	2.0
KENR77	*Streptomyces* sp.			n.d.	<0.5
KENR79	*Streptomyces* sp.			n.d.	1.0
KENR80	*Streptomyces* sp.			n.d.	15.6
KENR81	*Streptomyces* sp.			n.d.	3.9
KENR84	*Isoptericola* sp.			n.d.	3.9
KENR86	*Streptomyces* sp.			n.d.	500
KENR91	*Streptomyces* sp.			n.d.	7.8
KENR93	*Microbacterium testaceum*			n.d.	7.8
KENR94	*Streptomyces* sp.			7.8	n.d.
KENB3	*Streptomyces* sp.			n.d.	7.8
KENB8	*Streptomyces* sp.			n.d.	500
KENB9	*Streptomyces* sp.			n.d.	3.9
KENB10	*Microbacterium testaceum*			1000	250
KENS2	*Microbispora bryophytorum*			n.d.	2.0
n.d. – Not determined.			No Halo <1 cm	1–2 cm >2 cm	

*Diameter of the inhibition halos obtained by the agar disk diffusion method and MIC (minimum inhibitory concentration) values are shown.*

**FIGURE 4:**
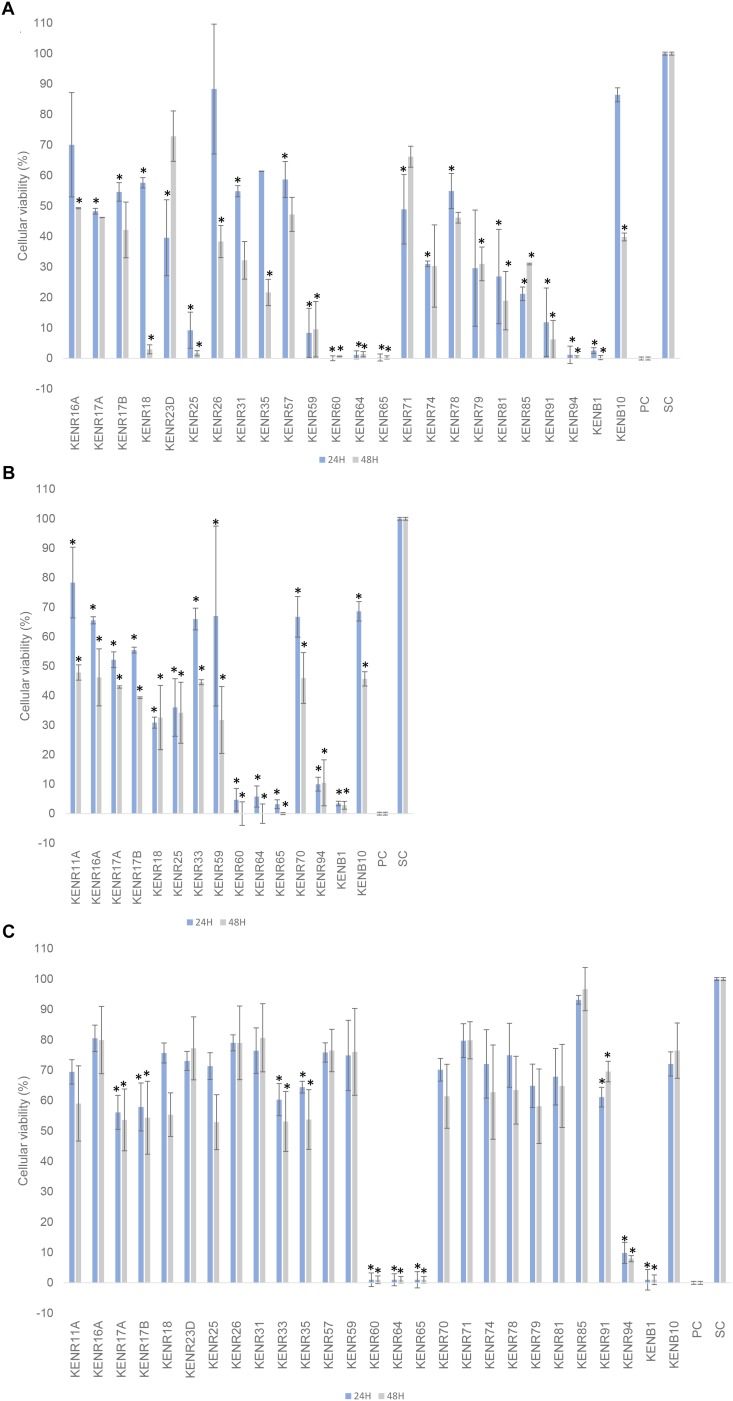
The effect of extracts from actinobacterial strains isolated from *L. ochroleuca* on cellular viability. The effects on the viability of **(A)** breast carcinoma T-47D and **(B)** neuroblastoma SH-SY5Y tumor cell lines, and **(C)** a non-tumor cell line (human brain capillary endothelial cells, hCMEC/D3) are shown after 24 and 48 h of exposure. Only the extracts with significant differences are shown on the graphs for SHSY-5Y and T47D cell lines. All of these extracts were re-tested on the non-cancer cell line **(C)** for general cytotoxicity. PC and SC indicate positive and solvent controls, respectively. Data are presented as mean ± standard deviation from two independent experiments performed in triplicates each, and significant differences compared to the solvent control are marked with asterisks in the graphs (^∗^ = *p* < 0.05).

Twenty-eight isolates showed statistically significant cytotoxic activity against at least one of the tested human cancer cell lines compared to the solvent control. From these, 24 isolates decreased the viability of SH-SY5Y cells, 15 decreased the viability of T-47D cells (Figure 4A,B) and 10 of the non-tumor cell line (Figure 4C). Strains KENR18, 25, 59, 60, 64, 65, 91, 94, and KENB1 (all *Streptomyces*) reduced the viability by more than 90% of the cell line SH-5YSY. Some of these strains (KENR60, 64, 65, 94, and KENB1) also caused a similar effect in the cell line T-47D and in the hCMEC/D3 cell line. Extracts with high cytotoxicity on hCMEC/D3 cells indicated a more general cytotoxicity. Here, we searched for strains that had cytotoxic activities toward human cancer cell lines, but not against the normal cells. In particular, extracts from the strains KENR18, 25, 59, 81, and 85 showed strong inhibition of SHSY-5Y cells (>80% inhibition), but without significant activity on normal hCMEC/D3 cells. This same pattern, albeit with lower potencies, was observed for the extracts obtained from strains KENR23D, 26, 31, 57, 71, 74, 78, and 79. The strains KENR18, 25 and 59 also reduced the viability of exposed T47D cancer cells (>70%), but not that of normal endothelial cells.

**FIGURE 5:**
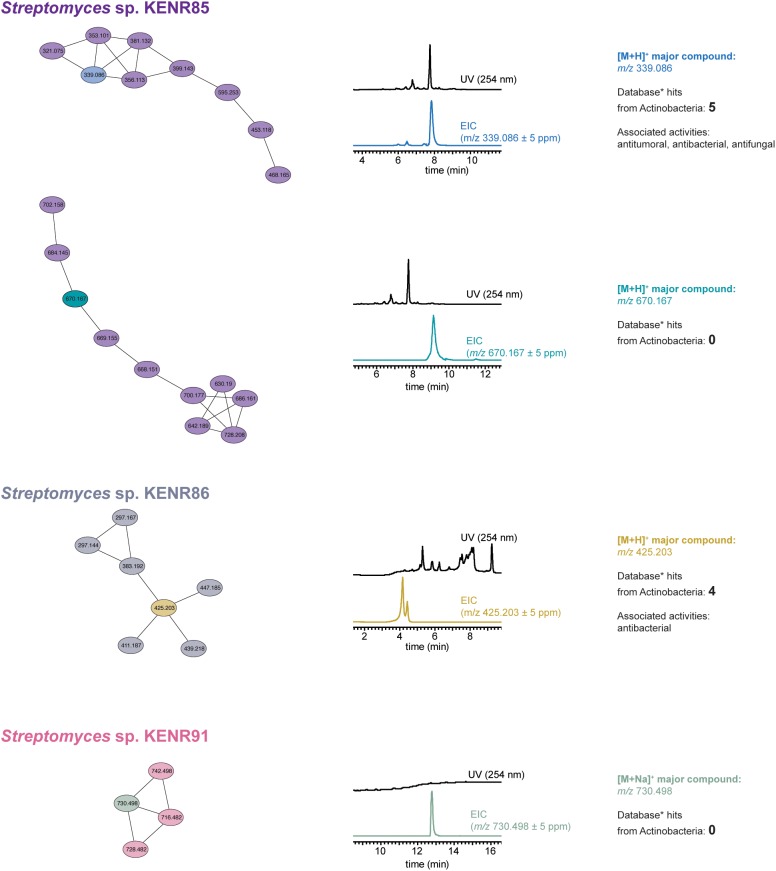
GNPS-based LC-HRESIMS/MS molecular network analysis of actinobacterial extracts likely to contain new bioactive compounds. The extracts obtained from three *Streptomyces* sp. strains showed molecular clusters unique to each strain (depicted by connected ellipses, labeled with the corresponding *m/z* value for the parent ion). The results of Dictionary of Natural Products (^∗^) searches for the accurate masses of the most abundant species in each cluster (as estimated from the respective UV and extracted ion chromatograms, EICs, and shown in different color from the remaining nodes in the cluster) are presented for each *m/z* species (for two molecular clusters, no correspondence was found in this database).

To understand whether some of the observed activities could be attributed to known compounds present in the tested samples, we selected 35 extracts that showed either strong antimicrobial or strong cancer cell line cytotoxicity and performed LC-HRESIMS/MS-based dereplication analysis using GNPS. Interestingly, we found that 26 of these extracts contained one or more members of the antimycin family of compounds (Supplementary Table S2). LC-HRESIMS/MS data for the remaining nine extracts (which showed no hits consistent with the observed activities in the GNPS dereplication), all from *Streptomyces* sp., were used to construct a molecular network in GNPS (Quinn et al., 2017) (Figure 5 and Supplementary Figure S2). This allowed us to pinpoint four clusters that were observed in a single extract, which we defined as an additional criterium for an increased chance of a cluster corresponding to a novel compound. Two of such clusters were found in the extract obtained from *Streptomyces* sp. KENR85, one from *Streptomyces* sp. KENR86 and one other from *Streptomyces* sp. KENR91. Because GNPS dereplication relies on the completeness of its database for MS/MS data matching, it is possible that previously NP do not show a hit in the GNPS dereplication workflow. As such, for each of these four clusters of compounds, we carried out an additional dereplication. We inferred the likely mass of the main compound from the corresponding LC-ESI-HRMS data and used that accurate mass as query in a search against the Dictionary of NP database. For two of the clusters, a number of hits from Actinobacteria were retrieved (Supplementary Table S3) that could explain the accurate mass and the observed activities in the corresponding extract. However, for the remaining two clusters (one from *Streptomyces* sp. KENR85 and one from *Streptomyces* sp. KENR91), no hits were retrieved from this database.

## Discussion

Macroalgae offer a favorable habitat for several epiphytic and endophytic microorganisms (Egan et al., 2013). Although actinobacterial endophytes of terrestrial plants have been proven to be a valuable source of bioactive compounds (Strobel, 2003; Zhao et al., 2011), their presence and diversity in macroalgae and their biotechnological potential is largely unknown. We isolated a high number of actinobacterial strains (some of them possibly endophytes) from the kelp *L. ochroleuca*, indicating that it is a rich reservoir of actinobacteria. The majority of the isolated strains was obtained from the holdfast of *L. ochroleuca*, which may reflect the permanent contact of this part of the algae with marine sediments and coastal rocks, where actinobacteria are known to predominate (Maldonado et al., 2005; Abdelmohsen et al., 2014).

*Streptomyces* strains are commonly isolated and cultured under laboratory conditions and have been isolated from numerous marine ecological niches (Rashad et al., 2015; Xiong et al., 2015). Several *Streptomyces* strains with biological activity have been isolated from algae, including from species belonging to the class Phaeophyceae where *L. ochroleuca* belongs (Wiese et al., 2009; Braña et al., 2015; Ismail et al., 2018). In our study, most of the isolates recovered from *L. ochroleuca* were also *Streptomyces* strains; this genus is the most important source of bioactive molecules within the phylum Actinobacteria (Dharmaraj, 2010).

Rare actinobacterial genera are also gaining increasing attention in the field of NP discovery, owning to their underexplored metabolic potential (Subramani and Aalbersberg, 2012, 2013; Dhakal et al., 2017). We isolated strains affiliated with six rare actinobacterial genera – *Rhodococcus*, *Nonomuraeae*, *Microbispora*, *Isoptericola*, *Nocardiopsis*, and *Microbacterium*. Isolates from the latter three genera have also been obtained from macroalgae species, including some from the class Phaeophyceae (Hollants et al., 2013; Martin et al., 2014; Santhi et al., 2014; Wang et al., 2017).

Half of the isolates exhibited antimicrobial activity, mostly antifungal activity against *C. albicans*, with four *Streptomyces* strains exhibiting the strongest activities. *Streptomyces* strains isolated in other studies from diverse marine sources have produced secondary metabolites with antifungal properties against *C. albicans*, like isoikarugamycin, a novel polycyclic tetramic acid macrolactam produced by *Streptomyces zhaozhouensis* (Lacret et al., 2014), or saadamycin, a polyketide produced by a marine sponge-associated *Streptomyces* sp. (El-Gendy and El-Bondkly, 2010). In this study we have also isolated eight actinobacterial strains exhibiting antibacterial activity against *S. aureus*, a bacterium associated with an extensive range of clinical infections, and with resistance to antibiotics, and an important target for the development of novel therapeutic agents (Tong et al., 2015). Marine actinobacteria are known to produce secondary metabolites capable of inhibiting the growth of *S. aureus*, including multidrug resistant strains of this bacterium. The compounds arenimycin and abyssomicin C are two such examples, produced by *Salinispora* and *Verrucosispora* strains, respectively (Bister et al., 2004; Asolkar et al., 2010). Recently, an actinobacterial strain identified as *Kocuria marina* CMG S2, isolated from the brown macroalgae *Pelvetia canaliculata*, was reported to produce a novel antibiotic, named as kocumarin, with activity against fungi and pathogenic bacteria, including methicillin-resistant *S. aureus* (Uzair et al., 2018).

Likewise, several actinobacterial strains isolated from different marine sources, including macroalgae, are capable of producing secondary metabolites with anticancer activity (Lam, 2006; Asolkar et al., 2010; Tong et al., 2015). Many of these strains are *Streptomyces* but genera exclusive from marine environments like *Salinispora*, *Salinibacterium*, and *Marinispora* are also considered promising sources of anticancer molecules (Lam, 2006; Zotchev, 2012). In our study, a total of 28 extracts demonstrated capacity to decrease significantly the viability of the cell lines. Many of those extracts demonstrated cytotoxicity also on the non-carcinogenic cell line hCMEC/D3, which indicates general cytotoxicity. However, some of the extracts with strong anticancer activity had negligible toxicity to non-tumor cells, and showed specificity toward neuroblastoma cells (KENR18, 25, 59, 91).

The bioassay data that we obtained indicated the presence of active compounds in the extracts and, hence, the potential for the discovery of new bioactive metabolites. However, a common issue in current NP discovery efforts (in particular those using actinobacteria) is the re-isolation of previously reported secondary metabolites, leading to a waste of time and resources (Gaudêncio and Pereira, 2015). As such, and to understand to what extent the observed bioactivities could result from known compounds and, concomitantly, which extracts were more likely to harbor new bioactive metabolites, we performed dereplication and molecular networking analyses. We found that a number of different antimycins was associated with many of the dereplicated samples. These likely antimycin-containing extracts were obtained from different genera of actinobacteria and from all sampled *L. ochroleuca* parts. Members of this class of metabolites have been isolated previously from marine actinobacteria (Imamura et al., 1993; Xu et al., 2011; Han et al., 2012), suggesting that marine environments selects for this chemical scaffold, a finding that might deserve further study. The molecular network analyses revealed that two of these strains – KENR85 and KENR91 – were potential producers of novel bioactive compounds. UV-Vis and ion chromatograms extracted from the LC-HRESIMS data indicate that these potential novel metabolites are not likely to be abundant in crude extracts (Figure 5), but we intend to follow-up on these interesting results. Moreover, we only analyzed in depth clusters from the molecular network that were associated with a single extract, which does not rule out the possibility for additional chemical novelty in the set of nine strains that showed no relevant hits in the GNPS dereplication.

## Conclusion

This work adds to the knowledge available on actinobacteria associated with marine macroalgae and their bioactive potential. Our data indicates that the kelp forest alga *L. ochroleuca* is a valuable source for the isolation of actinobacteria, both in terms of abundance and diversity, and a reservoir of NP with antimicrobial and anticancer bioactivities.

## Author Contributions

MG, PL, and MC designed and oversaw the whole study. MG and IR carried out the isolation of actinobacteria and taxonomic identification. MG and TR carried out cell line cytotoxicity assays. IA performed the kelp sampling. FP oversaw the molecular biology methodologies and performed the phylogenetic analysis together with MG. RU oversaw cell line assays and performed the statistical analysis. MG carried out the antimicrobial assays, LC-MS, and molecular networking/dereplication. MG and MC led the writing of the manuscript with contributions from all the co-authors.

## Conflict of Interest Statement

The authors declare that the research was conducted in the absence of any commercial or financial relationships that could be construed as a potential conflict of interest.

## Footnotes

1 https://blast.ncbi.nlm.nih.gov/Blast.cgi

2 http://www.ezbiocloud.net/

3 https://rdp.cme.msu.edu/index.jsp
