# Supplementary material for: Actinobacteria Isolated From Laminaria ochroleuca: A Source of New Bioactive Compounds
Source: Front Microbiol. 2019 Apr 9;10:683. doi: 10.3389/fmicb.2019.00683 (PMC6465344; doi:10.3389/fmicb.2019.00683)
Supplement: Supplementary file 1 [file Data_Sheet_1.pdf]

Supplementary Material

Actinobacteria isolated from *Laminaria ochroleuca:* a source of new bioactive compounds

Mariana Girão[1,2], Inês Ribeiro[1], Tiago Ribeiro[1], Isabel C. Azevedo[1], Filipe Pereira[1], Ralph Urbatzka[1], Pedro N. Leão[1], Maria F. Carvalho[1*]

[1] Interdisciplinary Centre for Marine and Environmental Research, University of Porto, Porto, Portugal
[2] Department of Biology, Faculty of Sciences, University of Porto, Porto, Portugal

Corresponding author*

M.F. Carvalho

CIIMAR – Interdisciplinary Centre of Marine and Environmental Research, University of Porto

Terminal de Cruzeiros do Porto de Leixões, Avenida General Norton de Matos s/n, 4450-208 Matosinhos, Portugal

e-mail: mcarvalho@ciimar.up.pt

Tel.: +351 223401804

Table S1. Taxonomic identification of the actinobacterial isolates recovered from *Laminaria ochroleuca* and corresponding GenBank accession number.

Isolate	Part of *L. ochroleuca* from which it was isolated	Taxonomic Identification		
		Closest Relative	Similarity (%)*	GenBank Acession Number
KENR1	Holdfast	*Streptomyces aureus*	99	MK254613
KENR3	Holdfast	*Streptomyces olivochromogenes*	99	MK254601
KENR4	Holdfast	*Streptomyces olivochromogenes*	99	MK254600
KENR5	Holdfast	*Streptomyces flavofuscus*	99	MK254572
KENR6	Holdfast	*Streptomyces sampsonii*	99	MK254568
KENR7	Holdfast	*Streptomyces gougerotii*	100	MK254563
KENR8	Holdfast	*Streptomyces atratus*	99	MK254615
KENR10	Holdfast	*Streptomyces diastaticus*	99	MK254610
KENR11	Holdfast	*Streptomyces diastaticus*	100	MK254609
KENR11A	Holdfast	*Streptomyces champavatii*	99	MK254597
KENR13	Holdfast	*Streptomyces coelicolor*	100	MK254596
KENR13A	Holdfast	*Streptomyces champavatii*	99	MK254595
KENR13B	Holdfast	*Streptomyces exfoliates*	99	MK254594
KENR13C	Holdfast	*Streptomyces diastaticus*	99	MK254605
KENR14	Holdfast	*Streptomyces xiamenensis*	98	MK254606
KENR16A	Holdfast	*Streptomyces thermodiastaticus*	99	MK254552
KENR16B	Holdfast	*Streptomyces champavatii*	99	MK254593
KENR17A	Holdfast	*Streptomyces sampsonii*	99	MK254592
KENR17B	Holdfast	*Streptomyces champavatii*	99	MK254591
KENR18	Holdfast	*Streptomyces globisporus*	98	MK254590
KENR19	Holdfast	*Streptomyces champavatii*	99	MK254589
KENR21	Holdfast	*Streptomyces sampsonii*	99	MK254588
KENR21A	Holdfast	*Streptomyces exfoliatus*	100	MK254587
KENR23A	Holdfast	*Streptomyces exfoliatus*	100	MK254586
KENR23B	Holdfast	*Streptomyces achromogenes*	99	MK254620
KENR23C	Holdfast	*Streptomyces champavatii*	100	MK254585
KENR23D	Holdfast	*Streptomyces coelicolor*	99	MK254584
KENR24	Holdfast	*Streptomyces champavatii*	99	MK254583
KENR25	Holdfast	*Streptomyces sampsonii*	99	MK254582
KENR26	Holdfast	*Streptomyces cyaneofuscatus*	99	MK254581
KENR27	Holdfast	*Streptomyces gougerotii*	100	MK254580
KENR28	Holdfast	*Streptomyces atratus*	99	MK254617
KENR29	Holdfast	*Nonomuraea coxensis*	99	MK254624
KENR30	Holdfast	*Nocardiopsis prasina*	99	MK254627
KENR31	Holdfast	*Streptomyces camponoticapitis*	99	MK254579
KENR32	Holdfast	*Streptomyces achromogenes*	99	MK254619
KENR33	Holdfast	*Streptomyces lannensis*	99	MK254604
KENR34	Holdfast	*Streptomyces champavatii*	99	MK254578
KENR35	Holdfast	*Streptomyces sampsonii*	99	MK254577
KENR36	Holdfast	*Streptomyces champavatii*	99	MK254576
KENR38	Holdfast	*Streptomyces flaveolus*	99	MK254607
KENR39	Holdfast	*Rhodococcus erythropolis*	99	MK254623
KENR40	Holdfast	*Streptomyces champavatii*	99	MK254575
KENR41	Holdfast	*Streptomyces tendae*	99	MK254553
KENR42	Holdfast	*Streptomyces mirabilis*	99	MK254603
KENR45A	Holdfast	*Streptomyces hebeiensis*	98	MK254574
KENR47	Holdfast	*Streptomyces coelicolor*	100	MK254573
KENR49	Holdfast	*Streptomyces atratus*	99	MK254616
KENR50	Holdfast	*Streptomyces sanglieri*	99	MK254599
KENR51	Holdfast	*Streptomyces sanglieri*	99	MK254598
KENR52	Holdfast	*Streptomyces mirabilis*	99	MK254602
KENR55	Holdfast	*Streptomyces albospinus*	99	MK254571
KENR57	Holdfast	*Streptomyces sampsonii*	99	MK254570
KENR59	Holdfast	*Streptomyces olivaceus*	99	MK254569
KENR60	Holdfast	*Streptomyces coelicolor*	100	MK254567

KENR64	Holdfast	*Streptomyces olivaceus*	99	MK254566
KENR65	Holdfast	*Streptomyces pratensis*	99	MK254565
KENR69	Holdfast	*Streptomyces sampsonii*	99	MK254564
KENR70	Holdfast	*Nocardiopsis prasina*	99	MK254626
KENR71	Holdfast	*Streptomyces brevispora*	99	MK254611
KENR72	Holdfast	*Streptomyces aureus*	99	MK254612
KENR74	Holdfast	*Streptomyces coelicolor*	99	MK254562
KENR75	Holdfast	*Streptomyces tendae*	99	MK254561
KENR76	Holdfast	*Rhodococcus erythropolis*	99	MK254622
KENR77	Holdfast	*Streptomyces champavatii*	99	MK254560
KENR78	Holdfast	*Rhoococcus erythropolis*	99	MK254621
KENR79	Holdfast	*Streptomyces coelicolor*	99	MK254559
KENR80	Holdfast	*Streptomyces coelicolor*	99	MK254558
KENR81	Holdfast	*Streptomyces champavatii*	99	MK254557
KENR82	Holdfast	*Isoptericola chiayiensis*	98	MK254634
KENR84	Holdfast	*Isoptericola chiayiensis*	99	MK254633
KENR85	Holdfast	*Streptomyces diastaticus*	100	MK254608
KENR86	Holdfast	*Streptomyces coelicolor*	99	MK254556
KENR87	Holdfast	*Streptomyces xiamenensis*	99	MK254551
KENR89	Holdfast	*Streptomyces atratus*	99	MK254614
KENR90	Holdfast	*Nocardiopsis prasina*	98	MK254625
KENR91	Holdfast	*Streptomyces sampsonii*	100	MK254555
KENR92	Holdfast	*Streptomyces fulissimus*	99	MK254554
KENR93	Holdfast	*Microbacterium testaceum*	99	MK254630
KENR94	Holdfast	*Streptomyces champavatii*	99	MK254547
KENS1	Stipe	*Microbacterium testaceum*	98	MK254629
KENS2	Stipe	*Microbispora bryophytorum*	100	MK254628
KENB1	Blade	*Streptomyces atratus*	99	MK254618
KENB3	Blade	*Streptomyces shenzhenensis*	100	MK254550
KENB5	Blade	*Streptomyces atratus*	99	MK254549
KENB6	Blade	*Streptomyces sampsonii*	99	MK254548
KENB7	Blade	*Microbacterium testaceum*	98	MK254631
KENB8	Blade	*Streptomyces champavatii*	99	MK254546
KENB9	Blade	*Streptomyces coelicolor*	99	MK254545
KENB10	Blade	*Streptomyces sampsonii*	99	MK254632

*According to 16S ribosomal RNA (Bacteria and Archaea) database from NCBI BLAST.

Table S2. GNPS dereplication results for the 35 actinobacterial crude extracts selected, indicating the compounds recorded for each one and the correspondent cosine score, which indicates the similarity of two MS/MS spectra from 0 (totally dissimilar) to 1 (completely identical).

Strain	Taxonomic Identification	Compound	Cosine	*m/z* error ppm	Lib *m/z*
KENR6	*Streptomyces* sp.	Antimycin A_2	0.92	12	535.27
		Antimycin A_3	0.92	17	521.26
KENR8	*Streptomyces atratus*	Antimycin A_2	0.91	12	535.27
KENR11A	*Streptomyces* sp.	Antimycin A_2	0.93	12	535.27
		Antimycin A_3	0.91	17	521.26
KENR13	*Streptomyces* sp.	Antimycin A_2	0.92	12	535.27
		Antimycin A_3	0.92	18	521.26
KENR13A	*Streptomyces* sp.	Antimycin A_2	0.92	11	535.27
		Antimycin A_3	0.92	18	521.26
KENR13B	*Streptomyces* sp.	Antimycin A_2	0.91	13	535.27
		Antimycin A_3	0.91	18	521.26
		Antimycin A_1	0.85	2	549.28
		Antimycin A_4	0.85	2	507.23
KENR14	*Streptomyces iamenensis*	Antimycin A_2	0.91	12	535.27
KENR17A	*Streptomyces* sp.	Antimycin A_2	0.92	13	535.27
		Antimycin A_3	0.91	17	521.26
KENR18	*Streptomyces* sp.	Antimycin A_2	0.92	12	535.27
		Antimycin A_3	0.92	18	521.26
KENR21A	*Streptomyces* sp.	Antimycin A_2	0.92	12	535.27
		Antimycin A_3	0.92	18	521.26
KENR25	*Streptomyces* sp.	Antimycin A_2	0.92	13	535.27
		Antimycin A_3	0.91	18	521.26
KENR29	*Streptomyces* sp.	Antimycin A_2	0.91	12	535.27
		Antimycin A_3	0.90	18	521.26
KENR31	*Streptomyces* sp.	No match	-	-	-
KENR33	*Streptomyces lannensis*	Antimycin A_2	0.92	12	535.27
		Antimycin A_3	0.92	18	521.26
KENR35	*Streptomyces* sp.	Antimycin A_3	0.93	17	521.26
		Antimycin A_2	0.92	11	535.27
KENR49	*Streptomyces atratus*	Antimycin A_2	0.91	13	535.27
KENR59	*Streptomyces* sp.	No match	-	-	-
KENR60	*Streptomyces* sp.	No match	-	-	-
KENR64	*Streptomyces* sp.	No match	-	-	-
KENR65	*Streptomyces* sp.	No match	-	-	-
KENR72	*Streptomyces aureus*	Antimycin A_2	0.92	12	535.27
		Antimycin A_3	0.90	18	521.26
KENR74	*Streptomyces* sp.	Antimycin A_2	0.92	11	535.27
		Antimycin A_3	0.92	17	521.26
KENR77	*Streptomyces* sp.	Antimycin A_3	0.92	11	521.26
		Antimycin A_2	0.90	17	535.27
KENR80	*Streptomyces* sp.	Antimycin A_2	0.92	12	535.27
		Antimycin A_3	0.91	17	521.26
KENR81	*Streptomyces* sp.	Antimycin A_2	0.92	13	535.27
		Antimycin A_3	0.91	17	521.26
KENR84	*Isoptericola sp.*	Antimycin A_2	0.92	13	535.27
		Antimycin A_3	0.91	17	521.26
KENR85	*Streptomyces* sp.	No match	-	-	-
KENR86	*Streptomyces* sp.	No match	-	-	-
KENR91	*Streptomyces* sp.	No match	-	-	-
KENR94	*Streptomyces* sp.	Antimycin A_3	0.88	18	521.26
KENS2	*Microbispora bryophytorum*	Antimycin A_2	0.90	12	535.27
KENB1	*Streptomyces atratus*	No match	-	-	-
KENB3	*Streptomyces* sp.	Antimycin A_2	0.92	12	535.27
		Antimycin A_3	0.92	18	521.26
KENB9	*Streptomyces* sp.	Antimycin A_2	0.91	12	535.27
KENB10	*Streptomyces* sp.	Antimycin A_2	0.88	17	535.27

Table S3. Dictionary of Natural Products dereplication results for the clusters selected for strains KENR85 and KENR91.

Strain	Compound	Molecular Formula	m/z Error ppm	Biological Activity	Biological Source
**KENR85**	WS 5995B	$C_{19}H_{14}O_6$	0.9	Antifungal	*Streptomyces auranticolor* P5365
	Fluostatin A	$C_{19}H_{14}O_6$	0.9	Antimicrobial	*Micromonospora rosaria* N160
	Landomycin A	$C_{19}H_{14}O_6$	0. 9	Antibiotic	*Streptomyces cyanogenus* S-136
	Tetrangomycin	$C_{19}H_{14}O_6$	0.9	Antimicrobial	*Streptomyces olivaceus* *Streptomyces matensis*
	Thiazostatin A	$C_{15}H_{18}N_2O_3S_2$	8.3	Antioxidant	*Streptomyces tolurosus*
**KENR86**	JBIR 107	$C_{24}H_{28}N_2O_5$	9.6	Antibiotic	*Streptomyces tateyamensis* NBRC 105047
	Streptophenazine A	$C_{24}H_{28}N_2O_5$	9.6	Antibacterial	*Streptomyces* sp. HB202 *Streptomyces* sp. BCC21835
	Streptophenazine B	$C_{24}H_{28}N_2O_5$	9.6	Antibacterial	*Streptomyces* sp. HB202 *Streptomyces* sp. BCC21835
	Streptophenazine G	$C_{24}H_{28}N_2O_5$	9.6	Antibacterial	*Streptomyces* sp. HB202

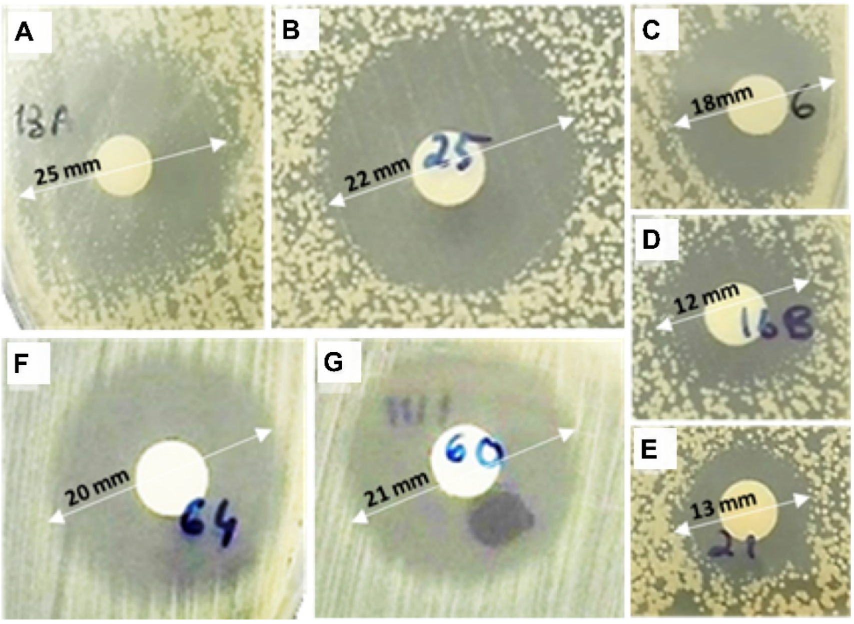

Figure S1. Antimicrobial activity of actinobacterial strains isolated from *L. ochroleuca*. (A-E) Examples of inhibition halos against *C. albicans* and (F-G) against *S. aureus*. (A) Strain KENR13A, (B) strain KENR25, (C) strain KENR6, (D) strain KENR16B, (E) strain KENR21, (F) strain KENR64 and (G) strain KENR60.

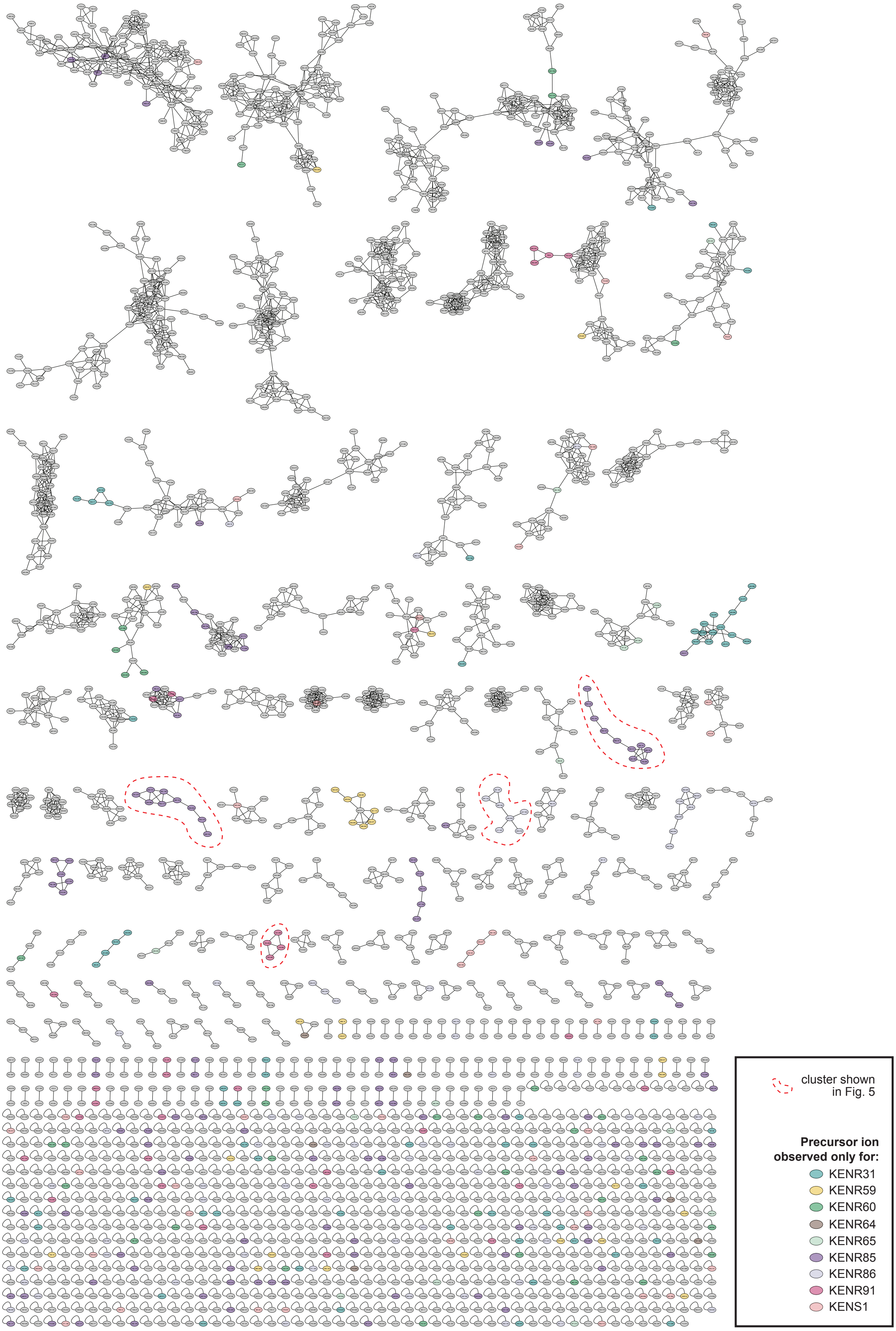

Figure S2. GNPS molecular networking using MS/MS data from extracts KENR31, KENR59, KENR60, KENR64, KENR65, KENR85, KENR86, KENR91 and KENB1. The value indicated in each node corresponds to the precursor ion.
